# Phobic memory and somatic vulnerabilities in anorexia nervosa: a necessary unity?

**DOI:** 10.1186/1744-859X-4-15

**Published:** 2005-09-06

**Authors:** Michael Myslobodsky

**Affiliations:** 1 Howard University and Cerebral Brain Disorder Branch, NIMH, NIH, Bethesda, MD 20892-1379, USA

## Abstract

Anorexia nervosa is a clinically significant illness that may be associated with permanent medical complications involving almost every organ system. The paper raises a question whether some of them are associated with premorbid vulnerability such as subcellular ion channel abnormalities ('channelopathy') that determines the clinical expression of the bodily response to self-imposed malnutrition. Aberrant channels emerge as a tempting, if rather speculative alternative to the notion of cognitively-driven neurotransmitter modulation deficit in anorexia nervosa. The concept of channelopathies is in keeping with some characteristics of anorexia nervosa, such as a genetically-based predisposition to hypophagia, early onset, cardiac abnormalities, an appetite-enhancing efficacy of some antiepileptic drugs, and others. The purpose of this article is to stimulate further basic research of ion channel biophysics in relation to restrictive anorexia.

## Introduction

Anorexia nervosa (AN) is an intractable illness with a high long-term mortality that affects 1% to 3.7% of the young women [1]. The death rate of patients with AN is up to 30 times greater than that of age-matched normal women. About 20% of AN patients remain chronically disabled [2]. Despite its grave complications, the lack of exact pathophysiology and organic definition, denies AN an independent nosological grade or a status of a neuropsychiatric problem. The various theories that have been proposed to explain the cause or origin of AN can be divided into two major schools of thought, socio-cultural and biological. Until very recently, AN was categorized as a disease of psychogenic origin (e.g., a consequence of unresolved conflicts of the individual psychosocial development) [3]. Many subscribed to the cultural paradigm inasmuch as it was rendered secure from experimental scrutiny. Not surprisingly, pharmacotherapeutic options in AN continue to be limited. For years, the disorder was so refractory that even 'heroic' efforts such as lobotomy, once reserved for treating schizophrenia were attempted [4]. Kennedy and Goldbloom [5] maintained in their review of 1991 that there was little, if any role for pharmacotherapy in AN. Over a decade later and more than 200 studies on the topic, the majority of patients stay refractory to the currently available drugs [6,7]. Therefore, alternative approaches toward AN pathophysiology has to be explored. A small proportion of individuals developing AN and the commonality and influence of socio-cultural pressures only emphasize the need for identifying the vulnerable population.

In keeping with this goal, the present article looks at the possibility that AN is associated with intrinsic vulnerability of potassium channels. It is to these channels and to their kinetics that cerebral cells owe their membrane potentials and the many characteristics that control local and distant changes in multiple organ systems. The following provides a selective perspective dealing mainly with the apamin-sensitive small-conductance calcium (Ca^2+^)-activated K^+ ^channels (SK channels) [8] as they might be related to the cognitive and somatic manifestations of AN.

### "Laparophobia" and K^+ ^channels

Young women with AN are recognized for commonly manifesting greater levels of general inhibition, loneliness, and social phobia of being corpulent. Also, fears associated with inadequacies of sexual life are found for 80% of the AN patients even if their initial stages of heterosexual development seemed normal [9]. Problems with sexuality may trigger the onset of AN [10]. In tardive AN (developing after the age 30), the feared sexuality emerged as being of apparent etiological significance, much as it is with earlier onset AN [11]. Therefore, elsewhere, AN was represented as anticipatory anxiety of stoutness and frank fat-phobia (*'laparophobia'*) [12,13] mitigated by the efforts to obtain alternative (non-appetitive) reward, such as exemplified by the paradoxical euphoric state and hyperactivity,[14] symptoms of denial, lack of concern, and alleged satisfaction with their state [15-17].

In theory, the acquisition of fear could be reduced to plasticity changes associated with Ca^2+ ^influx through N-methyl-D-aspartate (NMDA)-receptor channels in response to environmentally or cognitively elicited alarm. Ca^2+ ^ions control a host of neuronal functions, such as transmitter release, excitability and synaptic plasticity. In laboratory environment, Ca^2+ ^effects are reproduced by repetitive input stimulation that elicits a long-lasting increment of synaptic strength known as long-term potentiation (LTP), a widely known model of learning and memory processes (e.g. in the hippocampus or amygdala), with which a great number of neurophysiologic memory studies are performed. In many neuronal cells, intracellular Ca^2+ ^fluxes are increased during and after an action potential that activate K^+ ^channels thereby producing long-lasting changes of conductance and thus lasting membrane hyperpolarization. Therefore, it is conceivable that activity-dependent changes of excitability could be achieved via nonsynaptic mechanisms. Mechanistically, K^+ ^channels are defined as the pore-forming primary transmembrane proteins that initiate cellular polarization by allowing K^+ ^flux down a concentration gradient. Calcium-sensitive K^+ ^conductances are known to play a major role in the modulation of NMDA-induced bursting and the spike afterhyperpolarization, so that dysfunctional K^+ ^channels may contribute in the scenario of a wide range of cognitive aberrations and AN ideation. Ultimately, the process of learning and the strength of associations will be determined by the intrinsic morphology and kinetics as well as the density and distribution profile of ion channels that are embedded in the same membrane of the dendrites and the cell body, which accommodate NMDA receptors [18]. They play distinct physiological tasks from fine tuning membrane excitability in response to sensory input, modulating locomotion and emotional behavior to the induction of synaptic plasticity entailed in memory and cognition, temporally integrated with circadian cues, as well as in the antinociceptive effect [19].

Three subtypes of slow Ca^2+^-activated K^+ ^(SK) channels (SK1, SK2, SK3) set off by submicromolar intracellular Ca^2+ ^concentrations have been cloned, which differed in their pharmacology and kinetics [8,20-22]. One of the interesting features of SK channels is that normally they reduce neuronal excitability, whereas being blocked by the peptidergic honeybee toxin, apamin, they robustly augment neuronal excitability. In hippocampal CA1 neurons, SK channels contribute to the afterhyperpolarization, affecting neuronal excitability, regulating synaptic plasticity and memory [23]. Using field potential recording in the CA1-region of rat hippocampal slices, Behnisch et al. [24] showed that LTP induced by a single 100 Hz tetanization was intensified by extracellular application of apamin in a concentration range of 1–200 nM. These changes in the sensitivity to apamin were hypothesized to serve a marker of memory state and potentially, memory disorders. In fact, intracerebroventricular injection of apamin appears to improve retention of an odor pair association in rats [25]. Likewise, apamin increased neuronal excitability and facilitated the induction of synaptic plasticity at Schaffer collateral synapses and hippocampal-dependent learning [26]. Mice treated with apamin demonstrated accelerated hippocampal-dependent spatial and nonspatial memory encoding. They required fewer trials to learn the location of a hidden platform in the Morris water maze and less time to encode object memory in an object-recognition task compared with saline-treated mice [27]. Blank et al. [28] found recently that SK3 channel transcript and protein were more abundant in hippocampi from aged mice (22–24 months) compared to hippocampi from young mice (4–6 months). They showed that age-related decrement in trace fear conditioning (a hippocampus-dependent learning task) is correlated with elevated expression of SK channels of the SK3 type in the hippocampus as well as with reduced LTP. The effect was reversed when treated with SK3 antisense oligonucleotides. The authors further suggested that increased hippocampal expression of SK3 channels in aged mice may represent a mechanism that contributes to age-dependent decline in learning and memory and synaptic plasticity. In the hippocampus, SK3 was found predominantly in the terminal field of the mossy fibers and in fine varicose fibers, thereby suggesting their presynaptic localization. Using high-resolution immunofluorescence analysis, one study [18] found that the SK3 clusters were precisely colocalized with the presynaptic marker synapsin and at close range (0.4–0.5 mum) from NMDA-receptors and PSD-95, but rarely associated with GABA_A_-receptor clusters. This arrangement is consistent with a view that SK3 is a presynaptic channel in excitatory hippocampal synapses, with no preference for glutamatergic or GABAergic postsynaptic neurons, and is probably involved in regulating neurotransmitter release.

The responsibility of SK3 channels for the medium, and possibly the slow components of an afterhyperpolarization current make them a candidate for dysregulation of cellular homeostasis in a variety of systems. They are abundantly present in brain regions implicated in AN, such as the hypothalamus, [29] the limbic system [30] and midbrain regions [31,32] The cardinal role of SK channels in regulating burst firing and rhythmic oscillatory activity appears to be needed for coding for reward-related events by dopaminergic midbrain neurons. The afterhyperpolarization in dopaminergic ventral tegmental area neurons was shown to determine their sensitivity to ethanol reward [33] and by extension, they could be as responsive to some corporeal and extracorporeal rewards. Perhaps, this mechanism may be relevant for locomotion reward in hyperactive AN patients [14]. The Ca^2+^-dependent activation of K^+ ^channels may be among other regulators of pacemaker activity of interneurons that govern the quasi-periodic repetition of group activities and EEG synchronization. The excitability of fast-spiking prefrontal interneurons may be regulated by dopamine via a voltage-independent 'leak' K^+ ^current and an inwardly rectifying K^+ ^current, thereby modulating pyramidal cell excitability [34]. It remains to be explored which neurons express their distinct subsets of SK channel subunits in specific areas and how they are related to changes of cellular functions translated to the diverse clinical features of AN along its course and co-morbidities.

### Somatic and cognitive aberrations: Two in one?

The literature is definitive about an increased risk of diseases in AN. However, whereas psychological and cognitive deficits are conceived of as a component of the AN syndrome, somatic abnormalities are rather attributed to self-imposed malnutrition than to general vulnerability associated with AN [2]. Only infrequently are, cognitive and somatic alterations discussed together albeit in the context of a more specific shortage, such as caused by insufficiency in poly-unsaturated fatty acids that could cause also cognitive abnormalities [35]. Studies that obtain retrospective histories of disorders that occur prior to the age of onset of AN are uncommon, and are limited to psychopathological findings [36]. Assuming that there is a role for SK channels in AN, the question is what somatic manifestations would parallel neuropsychiatric abnormalities?

#### Medical comorbidity of AN

Of the three SK channels, SK1 and SK2 are predominantly expressed in the nervous system, in cortical pyramidal cells; [37] the basal ganglia and limbic system; [26,38,39] dopaminergic (DA) midbrain neurons; [40] and supraoptic neurosecretory cells [41]. The SK3 protein is seen more diffusely scattered. It is expressed primarily in phylogenetically older brain regions [42], and is also distributed in some peripheral neurons as well as diverse bodily tissues [8]. That wide distribution of SK channels and the fundamental role of K^+ ^currents in controlling membrane excitability of diverse organ systems pose a question of their role in regulation and dysregulation of the functional state of bodily tissues, at least of ectodermal origin, concurrently with that of the central nervous system.

It is telling that AN is associated with an amazing rate of cardiac abnormalities (in up to 86% of the patients) such as electrocardiographic abnormalities, reduced left ventricular mass, a small heart on the chest X-ray, impaired myocardial performance, and others.[43,44] Mitral valve prolapse (MVP) is another common somatic signs of AN.[45,46] Abnormality of the mitral apparatus may be primary or more benign secondary that emerge as a consequence of reduced or abnormal ventricular dimensions due to weight lost [47]. It is not always certain what form of MVP are registered in AN. The possibility of primary MVP abnormality cannot be ruled out since it may be associated with some subtle generalized disorder coupled with peculiar skeletal abnormalities (e.g. pectum excavatum, scoliosis, loss of normal kyphosis of the thoracic spine), somewhat elongated arms and decreased breast mass [48] The breast develops from the anlage of ectodermal cells along the primitive mammary ridges 'milk lines' during the sixth week of gestation. Certain abnormalities of the growing breast such as breast asymmetry (difference of its form, position or volume), hypoplasia of one breast are common finding in normal adolescents [49].

Cardiac arrhythmias and the lengthening of the QT interval are frequently associated with AN.[2] Recurrent syncope and sudden death typically occur in AN during exercise or emotional upset, [50,51] so that it is more likely to be attributed to metabolic aberrations associated with malnutrition, dehydration, or hypoglycemia, or socially-triggered emotional distress. The neurogenic mechanisms are clearly implicated in many cases of cardiac arrhythmia and sudden death in AN. Critchley et al. [52] demonstrated the role of mental and physical stress challenges in a group of 10 out-patients attending a cardiological clinic. Using H_2_(^15^)O PET, they obtained a robust positive relationship between right-lateralized asymmetry in midbrain activity and proarrhythmic abnormalities of cardiac repolarization during stress. However, mental and physical stress merely exposed the presence of enhanced cardiac arrhythmic vulnerability such as deficient myocardial repolarization [53]. A greater risk for the development and recurrence of coronary heart disease in women with psychiatric disorders such as depression, panic disorder, and generalized anxiety disorder although commonly attributed to psychosocial factors [54] might also be contributed by proarrhythmic state unmasked by psychosocial stressors. Mutations in genes encoding sodium or potassium channels were shown to underlie the 'Long QT syndrome,' causing arrhythmias [50,55]. It is a frequent cause of syncope and unexpected death in children and young adults, mostly women. Although the syndrome is an autosomal-dominant genetic disorder of cardiac electrical repolarization, the QT interval at presentation is normal about 10% of the time and just borderline prolonged in another 30%, so that premorbid vulnerability may be difficult to establish, and it is hardly looked for as a sign anticipating AN.

A remarkable aspect of the cardiomyocytes is their property for 'memory' of the signal transduction mechanisms and cardiac repolarization. A case in point is a persistent or 'remembered' T-wave on ECG during periods of the previous abnormal QRS complex in the sequence of altered ventricular pacing and manifested during sinus rhythm. This repolarization contributed to by specific K^+ ^channels [56,57] was provocatively labeled as "cardiac memory" [58]. We do not know whether or not "memory" of somatic cells marks a problem that remains latent in manner of 'functional teratogenesis' until triggered centrally (e.g. [52]). Nor do we know to what extent environmental stressors could unmask general SK channels abnormalities of alveolar epithelial cells in the lung, mesenteric and pulmonary arteries, vascular smooth muscles, genitourinary and gastrointestinal smooth muscle cells. Alkon [59] not only set out the question, he also came up with a theory that aberrant channels must represent a systemic disorder in Alzheimer's disease that involves not just the brain but other tissues such as skin, blood, and olfactory mucosa, as well. A change in channel activity leading to a cataract brings together such distant disorders as schizophrenia and myotonic dystrophy [60]. Both carry an increased risk of cataract, regardless of whether it was due to abnormal gene expression or followed drug intervention.

The skin, including its specialized forms such as the retina derives their origin from the same progenitors around the third ventricle. Cutaneous and mucocutaneous changes are well documented to be among the early diagnostic pointers to AN. They include xerosis (71%), cheilitis (76%), bodily hypertrichosis (62%), periungual erythema (48%), gingival changes (37%), and nail changes (29%) [61] along with the thinner body hair and abundant pilosebaceous glands.[62,63] Some skin signs are part of 'vasospastic syndromes' with a range of manifestations from cold intolerance, altered thermoregulation to physical stimuli or emotional stress to Raynaud's phenomenon, [64] the redness, itching, and burning of the skin, particularly fingers, toes, heels, nose, and ears exposed to cold known as perniosis [65,66]. Vasospasm could trigger acute severe exacerbations due to thrombosis inasmuch as the platelets obtained from patients with AN or severe peripheral vascular disease appear to be hyperaggregable [67].

The eye is frequently involved in the vasospastic syndrome, and ocular manifestations of microvascular dysfunction include alteration of conjunctival vessels, corneal edema, retinal arterial and venous occlusions, and others [66]. A high incidence of ocular involvement in the form of episcleral capillary aneurysms and subconjunctival hemorrhages, reduced mean tear production, conjunctival squamous metaplasia [68] is another example of vasospasm in AN. The pathophysiology of 'vasospastic syndromes' is obscure. The failure of the endothelium-derived hyperpolarizing factor may be one of the players. The latter is operationally defined as the hyperpolarization and associated relaxation remaining after the inhibition of the synthesis of NO synthase and prostaglandins (ref. [69] for review) that is possibly another word for K^+ ^channels [70].

Significant osteoporosis affects over half of all women with AN [71,72]. Occasionally, fragile bones due to osteoporosis could lead even to fracture of ribs [73] and the sternum [74]. The mechanisms of bone loss in this condition are poorly understood. Although a low estrogen level is implicated, administration of estrogen alone has not been shown to prevent bone loss. Grinspoon et al.[72] hypothesized that administration of bone trophic hormone, insulin-like growth factor I (IGF-I), a nutritionally dependent hormone [75] that stimulates osteoblast function and collagen synthesis would increase bone turnover in young women with AN. They did obtain hypothesized increased markers of bone turnover in severely osteopenic women. However, IGF-1 may also be prenatally programmed. Infants whose mothers were exposed to peak sunshine during their first trimester were born significantly heavier than infants whose mothers experienced low levels of sunshine during the same period. Tustin et al. [76] attributed facilitated prenatal growth to high levels of IGF-1 due to sunshine exposure during early gestation. Epidemiological studies suggest an association between weight in infancy and skeletal size and the risk of osteoporosis in adulthood. A significant association was established between birth weight and adult bone mineral content at the lumbar spine and femoral neck [77]

In sum, prospective studies of children at risk for AN are missing to establish the presence of somatic anomalies long before the onset of eating disorder. However, the presence of the foregoing aberrations during intrauterine life, though subtle, may be fated to affect adult health trajectories. Collectively, these changes were designated over 40 years ago as "reproductive casualty." [78] Nowadays, the latter latent 'functional teratogenesis' is actively discussed as part of the "Barker hypothesis" that postulates that a number of dysfunctions undergo programming during embryonic and fetal life; that individuals with a completely normal phenotype at birth, may acquire diverse disorders in adolescence or adulthood [79-81].

#### Is calorie restriction all bad?

Many physicians encounter AN at the bedside, with the syndrome comprised of gonadal failure (a low estrogen state and amenorrhea), cardiovascular abnormalities, osteopenia, thinning of skin and skeletal muscle wasting that are typical of human aging. Therefore AN would be expected to be a harbinger of the premature frailty, increased susceptibility to aging-related disorders and decreased longevity. As it happens, AN is not totally harmful and in special circumstances may even be beneficial. The overall cancer incidence among women with AN identified in the population-based Danish Psychiatric Case Register during 1970–1993 was slightly reduced by a factor of 0.80 (95% confidence interval 0.52–1.18) below that of the general population.[82] In a sample of 7303 Swedish women hospitalized for AN prior to age 40 years (the Swedish Registries from 1965 to1998) there was a more robust decrease in breast cancer incidence compared with the general female population of comparable age. AN developing prior to the first birth followed by a subsequent pregnancy was associated with an even more pronounced reduction in risk [83].

How can this be? Although deficient transmembrane potassium traffic in some cells is troubling, it appears to be associated with an intriguing gain in having reduced physiological and pathological proliferation capacity and thus a diminished oncogenic potential [84]. A population-based retrospective cohort study of 208 Rochester residents who were monitored for up to 63 years since admission for AN found that long-term survival in AN patients did not differ from that expected for the community [85]. We have yet to learn whether or not AN mitigates malignancies in tissues other than breasts that are vulnerable to aging (e.g., colon, bladder) and reduces neuronal loss in neurodegenerative disorders. The role of caloric restriction in increasing longevity was repeatedly demonstrated in laboratory animals and lower organisms. Assuming that all essential nutrients are acquired, AN might be conceived of as the cheapest investment into defenses against infections and cancer, in general. However, AN-style starvation carries unacceptably high risk when a wider range of outcome variables is considered [86].

#### Side Effects of Anorectic Drugs: Pathophysiology Ex-Juvantibus

Using a preparation of isolated rat lungs, Belohlavkova and colleagues [87] compared the inhibitory effect of ritanserin, an antagonist of 5-HT2 receptors, on fenfluramine- and 5-HT-induced vasoconstriction. As expected, both 5-HT and fenfluramine caused significant increases in perfusion pressure. Ritanserin at a dose (10-7 mol/l) inhibited >80% of the response to 5-HT and reduced the response to fenfluramine by approximately 50%. A higher ritanserin dose (10-5 mol/l) completely abolished the responses to 5-HT but had no more inhibitory effect on the responses to fenfluramine. However, a pharmacological blockade of voltage-gated K^+ ^channel activity (by 4-aminopyridine) markedly potentiated the pulmonary vasoconstrictor response to fenfluramine but was without effect on the reactivity to 5-HT. Clearly, the pulmonary vasoconstrictor response to fenfluramine was only partly mediated by 5-HT receptors, inasmuch as the vasoconstrictor potency of the drug was elevated when the K+-channel activity was reduced or altered in transgenic (SK3-T/T) mice [88]. Fenfluramine was widely employed for the treatment of obesity and abandoned after it was noticed to cause the development of valvular heart disease, hypertension, stroke and digital or mesenteric ischemia [89-91]. Consequently, the possibility was entertained that some individuals may have had intrinsically low activity of K^+ ^channels ("channelopathy"), which, as Belohlavkova and colleagues [87] hypothesized, was not functionally obvious under usual conditions but may have become exposed by anorectic drugs. Although their findings suggest the presence of microvascular dysfunction of unknown origin the term *channelopathy *alludes to a group of disorders that include, other than congenital long QT syndrome mentioned above, also cyclic vomiting syndrome, neuromyotonia, episodic ataxia, abdominal migraine, and migraine headaches as well as many others have been mapped to chromosomal regions that are rich in ion channel genes [92-94]. This hunch, though highly speculative encourages henceforth to explore, whether or not AN shares some of its pathophysiology with channelopathies.

### Is Anorexia a "Channelopathy"?

Aberrant channels emerge as a tempting, if rather speculative alternative to the notion of synaptic modulation deficit in AN. There are several requirements to suspect the presence of anomalous channels in a given disorder:

• Symptoms implicating specific ion channel genes

• The timing of disease onset or deterioration in childhood or adolescence

• Episodic character of manifestations

• Involvement of more than one organ or system

Chandy et al. [95] found that the second (3-prime) CAG repeat was highly polymorphic in control individuals, with alleles ranging in size from 12 to 28 repeats. They tested for an association between the longer alleles of SK3 and these neuropsychiatric disorders. There was a statistically significant overrepresentation of longer alleles in schizophrenia patients and a similar albeit nonsignificant trend in bipolar disorder patients, thereby suggesting that mild variations in the length of the polyglutamine repeats might produce subtle alterations in channel function, and in neuronal behavior. Other groups [96,97], however, did not confirm this finding. The most persuasive evidence indicating an association between inherited disorders of ion channels and AN is the discovery of the gene encoding for the SK channel.[98,99] They support the assumption of a common vulnerability for 'functional psychoses' that may include AN.

Although infrequently (5%), AN may be associated with epilepsy [100]. Epileptiform abnormalities in the EEG are infrequently recorded in AN patients,[101] although their rate is likely to be significantly underestimated since EEG is not routinely examined in eating disorders. In early comprehensive studies of EEG in behavioral disorders of childhood, eating disorders were not even mentioned [102]. However, the absence of clear indices of epileptiform abnormalities is not a critical violation of the channelopathy criteria. The notion of channelopathy may well be expanded into the territory of neurodegenerative disorders, such as Alzheimer's disease, [59,103] which is infrequently associated with epileptiform phenomena. On the other hand, neurologic AN complications comprise the majority of those consistent with the presence of channelopathies: neuromuscular abnormalities (45%); generalized muscle weakness (43%); peripheral neuropathies (13%); headaches (6%); syncope (4%); diplopia (4%), and movement disorders (2%). All these complications have episodic character. Also, local cellular epileptiform activity in the limbic system, such as plateau-bursting type action potentials or global Ca^2+ ^signal (typical for some endocrine-cell-type), may be recorded with no overt manifestations of episodic behavioral disturbances. Thus, a relative deficit of SK channels or even their genetically engineered absence [104] may not yield overt phenotypic outcome, as SK3 overexpression would [28]. Nonetheless, it would subtly increase excitability, reduce the threshold for the induction of synaptic plasticity, and facilitates amygdala or hippocampus-dependent memory. On the background of abnormal temporolimbic machinery such changes of molecular plasticity may conceivably cause hormonal effects to be exaggerated or idiosyncratic, which would set a stage for phobias and obsessive-compulsive symptoms [105].

Cellular excitability changes may be also associated with a coerced movement of water to maintain osmolarity during cellular activity [106]. Water is transported by the aquaporins, a family of membrane proteins that function as water channels in many tissues including neurons, glial cells, astrocytic foot processes near or in direct contact with blood vessels and others. Therefore, a loss of K^+ ^homeostasis in the presence of sustained neuronal activation may follow that of aberrant water fluxes. The mechanism underlying the functional coupling between water transport and K^+ ^has yet to be elucidated. It was noticed, however, that a lasting compromise of cell volume constancy could contribute to a buildup of K^+ ^in the extracellular space and ultimately, set a stage leading to a chronically enhanced excitability and even epileptogenicity [107,108].

One might further posit that estrogen represents additional factor modulating excitatory neurotransmission (apparently via NMDA/AMPA receptors) in the hippocampus [109] Using whole-cell recordings in hypothalamic slices from ovariectomized female guinea pigs, Kelly et al. [110] showed that estrogen (17β-estradiol, E2) robustly augments the efficacy of α1-adrenergic receptor agonists in inhibiting SK currents in preoptic GABAergic neurons. An association between susceptibility for eating disorders and the gene encoding for β-estrogen receptor [99] suggests that a specific group of individuals would have increased AN severity specifically related to changes in hormonal profile. In sum, the notion of AN as a channelopathy is in keeping with the following characteristics of the disorder: its early onset, genetic liability as well as episodic somatic disorders such as cardiac and autonomic abnormalities. As is shown below, the efficacy of some antiepileptic drugs is apparently also consistent with this hypothesis.

### Epilogue

The physiological and pathophysiological effects of K^+ ^channels on cerebral and extracerebral functions are numerous. Their role suggests a paradigm of "channelopathy" that articulated a way of simplifying and explaining otherwise seemingly unrelated somatic and neuropsychological findings in AN. If we are to understand the pathophysiology of the disorder, knowledge of the triggers of instabilities in ion fluxes in specially designed prospective studies may be mandatory.

The major limitation of researching the problem is in the populations selected because of multiple AN phenotypes. All patients entered in the studies cited above are either referred for their severe dieting or somatic manifestations consequent to it; many of them are interesting cases reported for their unusual presentation, such as nausea, vomiting, abdominal pain, electrolyte disturbances, sleep disorders, orthostasis and others [111,112].

The basis for choosing a conceptual model of AN, other than its simplicity, is the capacity of the model to provide a common denominator, for both psychopathological profiles and somatic manifestations of the disorder as well as to suggest therapeutic choices. SK3 channels may be potential therapeutic targets for regulating brain excitability as well as alleviating somatic disorders associated with AN. Several selective ligands are already being explored for their ability to block SK channel or facilitate SK channel opening [21,113]. Somewhat facetiously, Iversen[114] admonished that "on average it takes around 30 years for a new scientific discovery to find its way to a new generally available therapy" (p. 1539). Although new technologies may greatly facilitate the progress of identifying potential therapeutic targets, some caution need to be exercised [115]. The "thirty year rule" may still apply in the area of ion channel biophysics. With this in mind, the presence of abnormal neuronal excitability in AN, behooves the research clinicians to the fact that achieving membrane stabilization, reducing action-potential firing, and controlling Ca^2+ ^fluxes in the neural and non-neural tissues can be accomplished by using drugs activating a GABA_A _receptor complex that results in opening of the Cl^- ^channel and influx of Cl^- ^ions thereby leading to hyperpolarization of the neuronal membranes. However, studies on GABA_A_-mediated inhibition in eating behaviors have yielded both orexigenic and anorexic effects depending on drug used. Another anticonvulsant to consider is ketamine [116]. In patients with a long history of eating disorder that were resistant to several forms of treatment, Mills and colleagues [117] attempted infusions of 20 mg per hour ketamine for 10 h along with 20 mg twice daily nalmefene as opioid antagonist. Nine of 15 patients examined in the study showed prolonged remission when treated with two to nine ketamine-infusion sessions at intervals of 5 days to 3 weeks. Admittedly, ketamine has limited therapeutic potential because of its adverse psychotomimetic side-effects. In this regard, memantine (1-amino-3, 5-dimethyl-adamantane), an uncompetitive NMDA receptor antagonist may be a better alternative by producing symptomatological improvement under conditions of tonic NMDA receptor activation. Preclinical studies showed that memantine can reduce the behavioral deficits produced by chronic stress [118] and enhance antidepressive effects of fluoxetine given in subtherapeutic doses [119].

## Abbreviations

AMPA, amino-3-hydroxy-5-methyl-4-isoxazol propionate; AN, restrictive anorexia nervosa; DA, dopamine; GABA, γ-aminobutyric acid; 5-HT, 5-hydroxytryptamine; IGF-I, insulin-like growth factor I; K^+^, potassium; LTP, long-term potentiation; NMDA, N-methyl-D-aspartate; SK, slow potassium channel.

## Contributors

Michael Myslobodsky is the sole contributor to this review.

## Funding

No financial assistance was received for the writing of this paper.

## Competing interests

The author(s) declare that they have no competing interests.
